# Neonatal bacterial meningitis: the elusive search for markers of severity

**DOI:** 10.1016/j.jped.2026.101567

**Published:** 2026-06-04

**Authors:** Pablo J. Sánchez, Antoniece S. Duncanson, Jackson T. Gamer, Darrah N. Haffner

**Affiliations:** a The Ohio State University College of Medicine, Center for Perinatal Research, Abigail Wexner Research Institute at Nationwide Children’s Hospital, Nationwide Children’s Hospital, Department of Pediatrics, Divisions of Neonatology and Pediatric Infectious Diseases, Columbus, OH, USA; b The Ohio State University College of Medicine, Nationwide Children’s Hospital, Department of Pediatrics, Division of Neonatology, Columbus, OH, USA; c The Ohio State University College of Medicine, Nationwide Children’s Hospital, Department of Pediatrics, Columbus, OH, USA; d The Ohio State University College of Medicine, Nationwide Children’s Hospital, Department of Pediatrics, Division of Neurology, Columbus, OH, USA

Neonatal bacterial meningitis remains one of the most serious infections in perinatal medicine [1,2]. Despite advances in neonatal intensive care and antimicrobial therapy, neonatal bacterial meningitis continues to be associated with a high risk of death and neurodevelopmental impairment [2]. Timely identification and appropriate antimicrobial therapy of neonates with bloodstream bacterial infection is key for prevention of invasion of the central nervous system, yet finding the optimal biomarker for neonatal sepsis has remained elusive [3,4]. Once central nervous system infection has occurred, defining the factors that identify those neonates at high risk of later neurodevelopmental sequelae has remained a central challenge in neonatal care. In this respect, and as suggested in the timely study by Zhang et al. [5], early identification of infants at risk for acute neurological complications may help guide management and possibly mitigate later adverse sequelae.

In their retrospective cohort study of 68 neonates with bacterial meningitis admitted to a tertiary care pediatric medical center in China from 2020 to 2025, Zhang et al. identified acute neurological complications in 20 (29%) neonates [5]. The specific acute neurological complications and their frequencies were encephalomalacia (18%), subdural effusion/empyema (12%), hydrocephalus (10%), brain abscess (6%), and ventriculitis (4%). These findings were based on clinical and neuroimaging (cranial ultrasound or magnetic resonance imaging [MRI]) criteria. Importantly, all neuroimaging scans were reviewed independently by two pediatric neuroradiologists who were blinded to clinical outcomes. The authors found that compared to neonates without acute complications, those who developed acute neurological complications were older at symptom onset (median, 14.5 vs. 7.5 days) and had higher rate of seizures (55%vs. 6%), elevated c-reactive protein (mean, 87.9 mg/L vs. 62.3 mg/L), cerebrospinal fluid (CSF) culture positivity (80%vs. 48%), group B streptococcal (GBS) infection (65%vs. 17%), and more frequent dexamethasone administration (50%vs. 21%). Multivariate logistic regression analysis identified seizures (OR 11, 95% CI: 1.98–60.5; *p* = 0.006) and GBS infection (OR 4.8, 95% CI: 1.07–21.5; *p* = 0.04) as independent risk factors for acute neurological complications.

Overall, and not surprisingly, seizures emerged as the strongest clinical predictor of acute neurological complications [5]. Occurrence of seizures during bacterial meningitis serves as a clinical marker of cerebral involvement indicative of cortical irritation, inflammatory parenchymal injury, evolving cerebral injury, or infarct [[6], [7], [8]]. All neonates diagnosed with bacterial meningitis should have continuous electroencephalography (cEEG) monitoring [9]. Neonates with suspected meningoencephalitis are at increased risk for seizures, many of which are electrographic without clinical correlate and would be missed by clinical observation alone [9]. Furthermore, the occurrence of seizures should always prompt performance of optimal neuroimaging that initially may consist of cranial ultrasonography to detect hydrocephalus, large abscesses, or hemorrhage that may require expedient neurosurgical intervention [10]. However, more subtle findings may be missed by cranial ultrasonography and ultimately a contrasted brain MRI is indicated. The latter is the best modality to determine the extent of brain injury as well as the occurrence of cerebral or extra-axial abscess [11]. In addition, venous phase angiography via MRI or computed tomography (CT) may be needed to exclude sinovenous thrombosis [12]. In the study by Zhang et al. [5], the number of neonates who underwent the specific neuroimaging procedures as well as the timing of those procedures is unclear and could have influenced the reported frequency of neurological complications. Unfortunately, neurodevelopmental assessments were not performed to further highlight the importance of detection of acute neurological complications.

Meningitis due to GBS also was independently associated with acute neurological complications. This aligns with the well-described severe neuroinvasive potential of GBS in neonatal disease [13], [14], [15]. Despite widespread intrapartum chemoprophylaxis, and as reported by Zhang et al [5], GBS remains a leading cause of neonatal sepsis and meningitis globally and continues to account for substantial neurologic morbidity [14]. Zhang and colleagues did not differentiate early from late-onset GBS disease. Early-onset cases potentially could have been prevented by maternal GBS screening and intrapartum chemoprophylaxis. In the United States, meningitis is more commonly seen with late-onset GBS infection; preventive strategies remain elusive and likely will require the development of an effective maternal GBS vaccination strategy [14]. The second most common organism in blood or CSF was *Escherichia coli* (25%, 15/61) which has become the most common early-onset pathogen in the United States, especially among preterm infants [16]. The few cases (*n* = 3; 15%) of *E. coli* meningitis resulting in acute neurological complications are surprising as this disease can be neurologically devastating. It is unclear whether early death could have resulted in selection bias as affected neonates would not have been enrolled. In addition, there were 19 (31%) other bacterial pathogens detected in blood or CSF but not specifically identified in the study. Nevertheless, the findings do reinforce an important principle: pathogen identity is not a secondary detail in neonatal meningitis — it is central to optimal treatment and development of acute complications, and likely prognosis as well.

At the same time, and as acknowledged by the authors, CSF cultures were sterile in 43% of neonates diagnosed with meningitis. The finding that 80% of neonates with acute neurological complications had a positive CSF culture compared with 50% of those without a complication brings into question the diagnosis of bacterial infection in some of these neonates. In addition to a positive CSF bacterial culture, the diagnosis of meningitis was based on positive blood culture and at least two abnormal CSF findings such as pleocytosis, low glucose, or elevated protein, or even negative bacterial detection from CSF and blood but with clinical signs suggestive of meningitis and abnormal CSF indices. Although neonates with intraventricular hemorrhage were excluded, as well as those who had detection of nonbacterial pathogens in CSF, the CSF indices often are unreliable when the performance of the lumbar puncture is traumatic. It is unclear how Zhang and co-authors took this into account in the evaluation of CSF abnormalities. The finding of a positive blood culture in a neonate in whom one is unable to evaluate the CSF oftentimes prolongs antibiotic therapy unnecessarily to cover possible central nervous system disease. At the same time, sterile CSF culture could represent early and appropriate treatment of central nervous system infection although the authors were not able to ascertain antibiotic administration before performance of the lumbar puncture in their study patients. The duration of bacterial CSF culture positivity and time to sterilization of CSF also were not provided as these impact acute and later neurological complications [17]. A lumbar puncture always should be part of the evaluation for neonatal sepsis as it informs optimal selection of antimicrobial therapy, antibiotic stewardship, acute complications, as well as neurodevelopmental outcomes. Once the CSF is culture-positive, a repeat lumbar puncture to document CSF culture sterilization helps guide duration of antimicrobial therapy especially in Gram-negative meningitis where the minimum duration of treatment is three weeks, or two weeks after the CSF bacterial culture is sterile, whichever is longer [1].

Zhang and co-authors do not report on the contribution of molecular testing for identification of a causative organism in the CSF. Given the importance of bacterial pathogen detection in CSF of neonates evaluated for sepsis and the limited pathogen detection in currently available meningitis/encephalitis multiplex panels, development of a neonatal CSF PCR platform that will detect an expanded array of neonatal pathogens such as *Klebsiella* sp., *Enterobacter* sp.*, Staphylococcus aureus, Ureaplasma* sp.*, Mycoplasma hominis,* and fastidious organisms (e.g. *Paenibacillus* sp.) must be a future endeavor.

The authors also report that a predictive model for acute neurological complications that incorporated both seizures and GBS infection demonstrated good discriminatory capacity with a Receiver Operating Characteristic area under the curve of 0.814. However, the reported sensitivity of 55% was modest. Nearly half of infants who developed acute neurological complications would not be identified using the model alone. The high specificity of 94% suggests potential value in identifying a subgroup of neonates who are unlikely to have central nervous system complications. Importantly, they also reported no protective effect of dexamethasone on the development of acute neurological complications.

Dexamethasone as adjunctive treatment for bacterial meningitis has been shown to improve neurologic outcomes and possibly lower mortality in infants and children beyond the neonatal period [18]. Similar data are lacking in neonates, and its use is not recommended in this population. Yet, in the study by Zhang and colleagues, 29% (20/68) of neonates received dexamethasone. While not statistically different, more neonates (50%; 10/20) who received adjunctive treatment with dexamethasone had a higher rate of acute neurological complications than those who did not receive it (21%; 10/48). Currently, data on the use of adjunctive therapy with dexamethasone for neonatal bacterial meningitis remain insufficient to support its routine use for neuroprotection [19]. It is hoped that the ongoing Better Outcomes in Babies with Bacterial Meningitis (BOBBi) trial (https://www.npeu.ox.ac.uk/bobbi) being conducted in the United Kingdom and Canada will answer the question whether administration of dexamethasone to infants < 3 months of age with suspected or confirmed bacterial meningitis improves survival without moderate or severe neurodevelopmental impairment at 24 months corrected age.

In conclusion, Zhang and co-authors should be commended for highlighting the acute neurological complications of neonates who survive neonatal meningitis. Preventive strategies are urgently needed [3], [20]. In the meantime, early identification of infected neonates and timely institution of appropriate antimicrobial therapy are key to prevent acute and long term outcomes [20]. Future work should move beyond static prediction models and toward prospective, multicenter cohort studies that incorporate standardized definitions, early- versus late-onset infection stratification, appropriately targeted antimicrobial treatment, neuromonitoring and imaging, as well as longitudinal neurodevelopmental follow-up assessments. The enigma of preventing and optimally managing neonatal meningitis persists.

## Abbreviations

GBS, group B streptococcus; MRI, magnetic resonance imaging; CI, confidence interval; CSF, cerebrospinal fluid; cEEG, continuous electroencephalogram; OR, Odds Ratio; CT, computed tomography.

## Funding source

None.

## Data availability

N/A.

## Conflicts of interest

Pablo Sánchez received consultation fees from Merck Sharpe & Dohme, 10.13039/100004334Merck, Kamada Pharmaceuticals, and CyanVac LLC (Blue Lake Biotechnology) unrelated to this study. Other authors have nothing to declare.
